# A molecular approach integrating genomic and DNA methylation profiling for tissue of origin identification in lung-specific cancer of unknown primary

**DOI:** 10.1186/s12967-022-03362-2

**Published:** 2022-04-05

**Authors:** Kaiyan Chen, Fanrong Zhang, Xiaoqing Yu, Zhiyu Huang, Lei Gong, Yanjun Xu, Hui Li, Sizhe Yu, Yun Fan

**Affiliations:** 1 The Cancer Hospital of the University of Chinese Academy of Sciences (Zhejiang Cancer Hospital), Hangzhou, 310022 China; 2 Institute of Basic Medicine and Cancer (IBMC), Chinese Academy of Sciences, Hangzhou, 310022 China; 3 Department of Thoracic Medical Oncology, Zhejiang Cancer Hospital, Hangzhou, 310022 China; 4 Department of Breast Surgery, Zhejiang Cancer Hospital, Hangzhou, 310022 China; 5 Department of Clinical Trial, Zhejiang Cancer Hospital, Hangzhou, 310022 China; 6 Department of Thoracic Medical Oncology, The Cancer Hospital of the University of Chinese Academy of Sciences (Zhejiang Cancer Hospital), Hangzhou, 310022 China

**Keywords:** Cancer with unknown primary (CUP), Multiple primary tumor, Comprehensive genomic profiling (CGP), DNA methylation, Machine learning, Lung cancer

## Abstract

**Background:**

Determining the tissue of origin (TOO) is essential for managing cancer of unknown primary (CUP). In this study, we evaluated the concordance between genome profiling and DNA methylation analysis in determining TOO for lung-specific CUP and assessed their performance by comparing the clinical responses and survival outcomes of patients predicted with multiple primary or with metastatic cancer.

**Methods:**

We started by retrospectively screening for CUP patients who presented with both intra- and extrathoracic tumors. Tumor samples from included patients were analyzed with targeted sequencing with a 520-gene panel and targeted bisulfite sequencing. TOO inferences were made in parallel via an algorithm using genome profiles and time interval between tumors and via machine learning-based classification of DNA methylation profiles.

**Results:**

Four hundred patients were screened retrospectively. Excluding patients definitively diagnosed with conventional diagnostic work-up or without available samples, 16 CUP patients were included. Both molecular approaches alone enabled inference of clonality for all analyzed patients. Genome profile enabled TOO inference for 43.8% (7/16) patients, and the percentage rose to 68.8% (11/16) after considering inter-tumor time lag. On the other hand, DNA methylation analysis was conclusive for TOO prediction for 100% (14/14) patients with available samples. The two approaches gave 100% (9/9) concordant inferences regarding clonality and TOO identity. Moreover, patients predicted with metastatic disease showed significantly shorter overall survival than those with multiple primary tumors.

**Conclusions:**

Genome and DNA methylation profiling have shown promise as individual analysis for TOO identification. This study demonstrated the feasibility of incorporating the two methods and proposes an integrative scheme to facilitate diagnosing and treating lung-specific CUPs.

**Supplementary Information:**

The online version contains supplementary material available at 10.1186/s12967-022-03362-2.

## Introduction

Lung cancer (LC) is the second most frequent cancer worldwide [1]. Approximately 50% of lung cancer patients present with distant metastases at diagnosis [2]. Meanwhile, the lung is a common destination for metastatic spread from other primary malignancies such as colorectal, cervical, and gastric cancer [3–6]. For patients presenting with intra- and extrathoracic tumors, it is paramount to elucidate the tumor relationship due to the therapeutic implications. Despite advances in conventional diagnostic work-up, histopathologically indeterminate cases still arise, constituting cancers of unknown primary (CUPs). CUP are typically characterized by dismal prognosis, with median overall survival of 1 year [7]. There is an unmet need for tissue of origin (TOO) identification for patients with lung-specific cancer of unknown primary (CUP).

A handful of studies have explored the value of different molecular tests for TOO identification [8–10]. Notably, gene expression profiles (GEPs) were used in a phase II trial that randomized CUP patients to standard chemotherapy and site-specific therapy guided by TOO inferred from GEP [11]. A later trial combined site-specific treatment based on GEPs and targeted therapy guided by genome profiles [12]. In both investigations, median overall survival (OS) and progression-free survival (PFS) were better for those predicted with more-responsive than with less-responsive tumor types, although no significant difference was observed for OS, PFS, and 1-year survival between site-specific and standard chemotherapy [11, 12]. In addition, there have been studies that used genomic alterations to distinguish multiple primary LC from intrapulmonary metastasis [13, 14] or DNA methylation to classify tumor type [15–17]. As a major epigenetic mechanism, DNA methylation refers to methylation generally of the cytosine base within the context of CpG dinucleotide repeats [18]. Features of DNA methylation patterns, such as hyper- or hypomethylation at certain sites and the relative ordering of relative methylation orderings of CpG sites, show marked tissue specificity [16, 19]. Despite encouraging advances, so far most investigations on TOO determination have focused on individual molecular analysis. Therefore, TOO identification and treatment of CUP patients may benefit from integration of different molecular tests.

In this study, we aim to establish an integrative approach for TOO inference based on multidisciplinary evidence. To this end, we retrospectively 16 patients with lungs-specific CUPs. The tumor origins were inferred from genome profiles and clinical information and from DNA methylation profiles, respectively. We then evaluated the level of concordance between these two methods and whether the predicted diagnoses were consistent with clinical outcomes. Furthermore, we propose an integrative molecular approach based on these results for TOO identification.

## Methods

### Patients

**Fig. 1:**
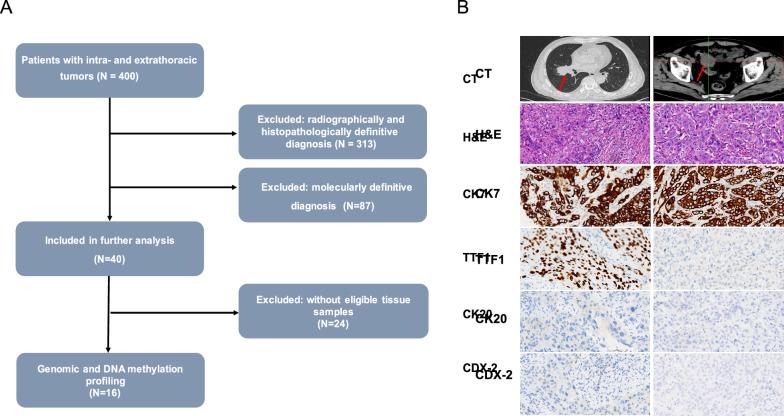
Study design. **A** A total of 400 patients with lung-specific CUPs were screened. Major exclusion criteria included definitive diagnosis by imaging, histopathologic, or molecular findings, inadequate tissue samples, and incomplete follow-up data. Sixteen patients were finally included in our study. CUP, cancer with unknown primary. **B** Representative findings from a case (P1) for which conventional diagnostic work-up did not reach a definitive diagnosis. Original magnification ×200. CT: computed chromatography. H & E: hematoxylin and eosin

We retrospectively screened for 400 patients who presented with intrathoracic and extrathoracic tumors and visited Zhejiang Cancer Hospital between March 2019 and January 2021. Extrathoracic tumors were defined as lesions other than those in the lungs and corresponding regional lymph nodes per the eighth edition of the tumor-node-metastasis (TNM) classification of lung cancer [20]. Eligibility criteria included a diagnosis of lung-specific CUP after comprehensive examination, including pathological evaluation by immunohistochemistry, chest-abdomen-pelvis computed tomography scans, and directed assessment of all symptomatic areas. Results of the laboratory tests, histopathologic work-up, and molecular and imaging studies were assessed by an institutional multidisciplinary team. Consensus regarding tumor clonality and origin was reached in 360 of 400 patients. As the flow chart in Fig. 1A indicates, excluding 24 patients with no eligible samples, 16 patients were finally included in this study. Differential diagnosis for all 16 patients was conducted using immunochemical staining with tumor type-specific markers, including CK20 and CDX-2 for colorectal, TTF1 and NapsinA for pulmonary, CK7 and CK20 for gastric cancer, and P16 for cervical cancer. Collection and analysis of tumor samples were approved by the Institutional Review Board of Zhejiang Cancer Hospital (No.IRB-2021-54). Informed consent was obtained from all patients.

### Comprehensive genomic profiling (CGP) analysis and inference of tumor relationship

For CGP, all 16 included patients provided one samples for the intrathoracic tumor and one for the extrathoracic tumors. A total 32 formalin-fixed and paraffin-embedded (FFPE) tissue specimens were subjected to targeted sequencing with a panel of 520 cancer-related genes (OncoScreen Plus, Burning Rock Biotech, Guangzhou, China). A sizable number of clinical investigations in multiple tumor types have conducted molecular testing using this panel, including lung cancer, colorectal cancer, and cervical cancer [6, 21, 22]. DNA extraction, quantification, library construction, sequencing, and data processing were performed as previously described [23]. A more detailed description is provided in the Additional file 1. The sequencing analysis detects single nucleotide variants (SNVs), small insertions and deletions, rearrangements, and splice variants. Tumor relationship was inferred via a multi-step algorithm. Multiple primary tumors were predicted if the tumor samples shared no genomic alteration and metastatic cancer was predicted if the tumors shared clinically actionable *EGFR* mutations or deletions or *ALK* or *ROS1* rearrangements. The remaining cases were assigned as “inconclusive as to tumor relationship” if they met neither of the following criteria: (i) both tumors harbored ≥ 5 genomic alterations and ≥ 2 of which were shared, or (ii) either tumor harbored < 5 alterations and ≥ 1 of which were shared. If any of these criteria was met, we further considered the time interval between manifestation of the tumors. For metachronous cases (detected at an interval of ≥ 6 months), the inferred diagnosis was metastatic disease originating from the organ involved in the earlier tumor, otherwise the case would be regarded as metastatic cancer but inconclusive as to TOO.

### DNA methylation profiling

A total of 29 samples were available for analysis, including paired samples of intra- and extrathoracic tumors from 14 patients and a sample of gastric tumor from one patient. DNA methylation profiling as performed as previously described [24]. Briefly, the bisulfite sequencing (BS-seq) library was prepared with the brELSATM method. Custom-designed methylation profiling RNA baits were used for target enrichment which covers 80,672 CpG sites and spans 1.05 mega base of human genome. The target libraries were quantified and sequenced on NovaSeq 6000 (Illumina, San Diego, CA, USA) with an average sequencing depth of 1000×. Further bioinformatic analyses were performed to remove custom adaptor sequences and low-quality bases, align and merge paired-end reads, and build methylation blocks. A more detailed description is provided in the Additional file 1.

### Construction of tissue classification models and TOO inference based on DNA methylation profiles

DNA methylation-based classifiers were constructed using a previously described machine learning approach [19]. Briefly, differential methylation sites were selected from The Cancer Genome Atlas database and further segregated into blocks, and the “block-level” methylation profile was represented with a matrix named “MBS”. A support vector machine classifier was implemented to construct classification models that categorized the tumor binarily as LC-origin or non-LC-origin. The algorithm maps training samples to points in a high-dimensional space. The width of the gap between two categories was maximized, and each mis-mapped training sample was penalized according to the parameter set to the model. A proprietary dataset was used for model training, which consisted of 70 tumor tissue samples from four sites, including 22 from lungs, 19 from stomach, 16 from colon, and 13 from cervix (Burning Rock Biotech, Guangzhou, China). Three classifications models were constructed to assign the tumor origin as the lung or one of the three alternative organs. In each model, LC and non-LC training samples were labeled with 0 and 1, respectively. The desired parameter was achieved through fivefold cross-validation using the training samples.

For TOO inference, DNA methylation profiles from the 29 samples were subjected to one of the three models based on the site of the extrathoracic tumor. Theses profiles were mapped into the same high-dimensional space in the identification models and assigned a Methyl Score based on the detected TOO-related methylation signals. As determined via the Youden index, the best cutoff (0.5) was applied to distinguish between LC-origin (Methyl Score < 0.5) and non-LC-origin (Methyl Score > 0.5) tumor tissue samples.

### Assessment of clinical response

Patients were evaluated for responses every 6 weeks after treatment onset until the detection of tumor progression or treatment completion, at which point they were all evaluated for survival outcomes. Tumor responses were assessed according to Response Evaluation Criteria in Solid Tumors, version 1.1.

### Statistical analyses

Statistical analyses were performed with the statistical programming language R and GraphPad Prism. Survival curves were estimated with the Kaplan–Meier approach and tested for significance using the log-rank test. Statistical significance was defined as P < 0.05 in a two-sided test.

## Results

### Patient characteristics

In this single-center retrospective study, we identified 400 patients with LC and a synchronous or metachronous tumor involving another organ. Despite thorough analysis and consultation with a multidisciplinary tumor board, 40 cases (10.0%) remained indeterminate due to inconclusive clinical, histopathologic, and imaging findings. Excluding 24 individuals without eligible tissue samples, matched tissues from 16 cases were analyzed for genomic aberrations and/or DNA methylation profiles (Fig. 1A). Figure 1B shows representative findings from a case for which multidisciplinary consulting did not reach a definitive diagnosis based on conventional diagnostic work-up. Clinicohistologic characteristics of these 16 patients are summarized in Table 1. The median age was 53 years, with a range of 47–77 years. Among them, 62.5% (n = 10) were male, 43.8% (n = 7) had a family history of cancer, and 31.3% (n = 5) were ever-smokers. Most patients had a good ECOG performance status of 1 (n = 12, 75.0%). The extrathoracic tumors were located in the stomach (n = 5), intestine (n = 7) and cervix (n = 4). According to the WHO classification for tumors, 68.8% (n = 11) of cases were identified as adenocarcinoma. The remaining five (31.2%) non-adenocarcinoma cases consisted of three poorly-undifferentiated carcinomas, one squamous carcinoma, and one atypical carcinoid.

**Table 1:** Baseline clinicopathologic characteristics of the patients included in this study

Characteristic	No. (%)
Age, median (range), y	53 (47–77)
Sex	
Male	10 (62.5)
Female	6 (37.5)
ECOG performance status	
1	12 (75.0)
2	4 (25.0)
Extrathoracic lesions	
Stomach	5 (31.2)
Colon/rectum	7 (43.8)
Cervix	4 (25.0)
Histology	
Adenocarcinoma	11 (68.8)
Squamous cell carcinoma	1 (6.3)
Poorly-undifferentiated carcinoma	3 (18.8)
Other	1 (6.3)
Smoking history	
Never-smoker	11 (68.6)
Ever-smoker	5 (31.3)
Family history of cancer	
No	9 (56.3)
Yes	7 (43.8)

### TOO inference based on comprehensive genomic profiling (CGP)

**Fig. 2:**
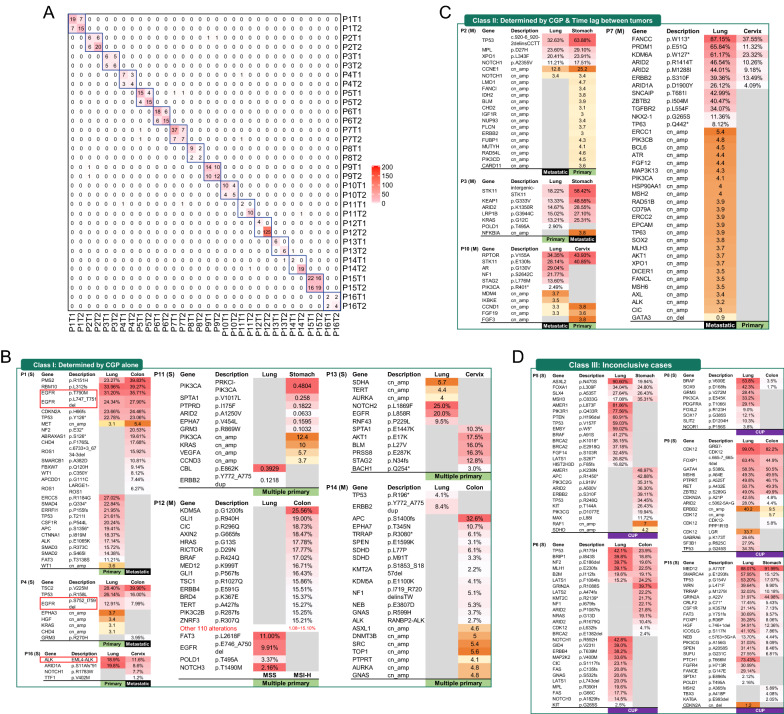
Tumor clonality and origin inferred from comprehensive genomic profiling (CGP) and inter-tumor time interval. **A** A heatmap showing the number of total genomic alterations identified in a tumor (in the form of row or column sum) and the number of those shared by the corresponding tumor pairs. T1 refers to intrathoracic tumors and T2 extrathoracic ones. **B**–**D** Profiles of genomic alterations for patients classified into three classes: **B** unambiguous TOO inference enabled by CGP alone, **C** unambiguous TOO inference enabled by CGP and inter-tumor time lag, and **D** inconclusive cases. All samples were microsatellite stable (MSS) except for the colon tumor of P12, which was microsatellite unstable (MSI)

CGP was performed on matched intra- and extrathoracic tumor tissues from all included 16 patients, followed by inference of tumor clonality and origin using a multi-step algorithm. As summarized in Fig. 2A, no alteration was shared between the matched samples for patients 11–14 despite the relatively large number of detected alterations (range of sum, 12–125). Detailed identities and abundances of these genomic aberrations are listed in Fig. 2B–D. Based on the presence of clinically relevant, high-specific driver alterations in LC, such as *EGFR* exon 19 deletion and *EML4-ALK* rearrangement, metastatic cancer with an LC origin was inferred for the corresponding patients 1, 4, and 16. As such, CGP data alone enabled unambiguous TOO identification for these 7 patients, who were grouped as Class I patients (Fig. 2B). The remaining 9 cases were first assessed for tumor relatedness based on the extent of profile similarity. Profile pairs that did not pass this test were categorized as inconclusive evidence. All 9 cases in this study met the clonality criteria and therefore diagnosed with metastatic cancer. Among these patients, patients 2, 3, 7, and 10 presented with metachronous tumors (detected at least 6 months apart) and their TOOs were inferred as the organ involved in the earlier tumor (Class II; Fig. 2C). The remaining 5 cases were also categorized as inconclusive (Class III; Fig. 2D). Altogether, CGP alone enabled unambiguous inference of tumor clonality for all 16 cases and TOO for 7 (43.8%), and the latter rate rose to 11 (68.8%) after integrating clinical evidence regarding time lag between the tumors.

### Construction of classification models and TOO inference based on DNA methylation profiling

**Fig. 3:**
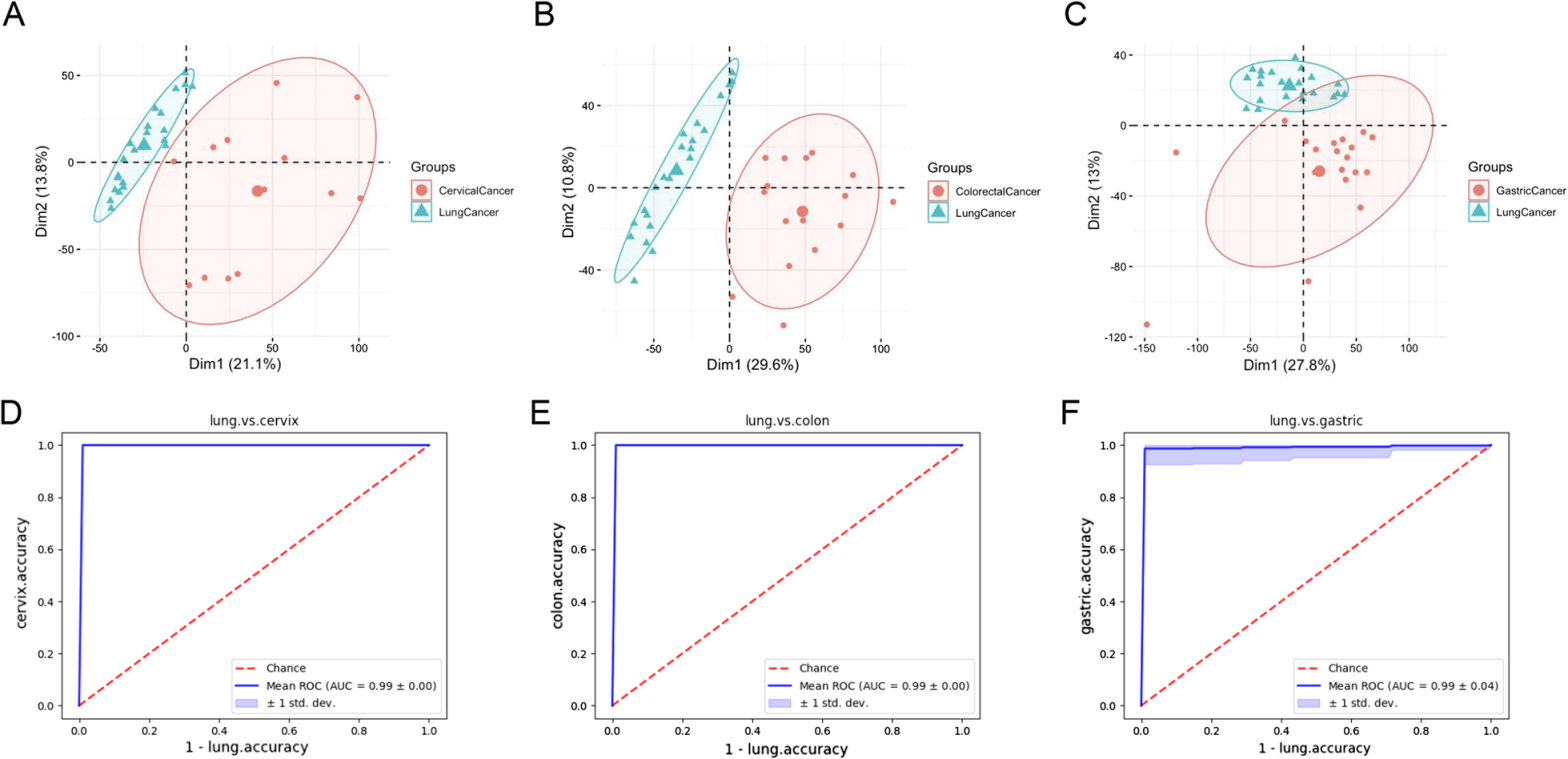
DNA methylation patterns from different disease sites and performance of classification models constructed with DNA methylation profiles. **A**–**C** Principal component analyses showing pairwise comparison of DNA methylation patterns between tumors from the lung and **A** cervix, **B** colon, and **C** stomach. **D**–**F** ROC curves of the TOO classification models based on methylation profiles showing remarkable model performance (AUC > 0.98). AUC: area under curve. ROC: receiver operating characteristic. TOO: tissue of origin

A machine learning approach was used to construct tissue classifiers based on DNA methylation signals detected from a training set of 70 tumor samples of known origins [19]. As tissue specificity has been established in DNA methylation patterns and serves as the basis for TOO identification [25], we tested the validity of our data by interrogating the distinctiveness of profiles from different organs. Indeed, principal component analysis indicated separate or largely non-overlapping clusters consisting mainly of samples from the same disease sites (Fig. 3A–C). Cross-validation suggested high performance of all three models in distinguishing tumors of LC versus non-LC origin, as indicated by the high areas under the curves (0.99 for all; Fig. 3D, F, Additional file 1: Figs. S1, and S2).

Of the 16 included patients, 14 had 2 DNA methylation profiles each, 1 patient (P11) had 1 available profile and 1 patient (P16) had none (Table 2). A total of 29 profiles were therefore which subjected to a site-appropriate classification model. As summarized in Table 2, DNA methylation analysis was able to unequivocally assign TOO for all 14 patients with methylation profiles from both tumors. Moreover, of the 9 patients with TOO inferred by both CGP-time lag and DNA methylation analyses, all had identical inferences. For P11 and P16, CGP was able to unambiguously assign TOOs for their tumor samples and matched the DNA methylation-based inference for the one sample from P11. In summary, DNA methylation profiling achieved a higher inference rate (100%) than the CGP-time lag approach (68.8%) and 100% agreement with latter in terms of TOO identity (9/9).

**Table 2:** Inferred tumor relatedness and origin for the 16 patients with lung-specific cancer of unknown primary

Patient ID	Time lag between tumors	Tumor location	Primary vs. metastatic lesions			CGP-methylation concordance
			Per CGP analysis	Per DNA methylation analysis	Methyl score	
1	Synchronous	Lung	P	P	0.475	Y
		Colon	M	M	0.378	
2	Metachronous	Lung	M	M	0.537	Y
		Stomach	P	P	0.634	
3	Metachronous	Lung	P	P	0.393	Y
		Stomach	M	M	0.354	
4	Synchronous	Lung	P	P	0.349	Y
		Colon	M	M	0.391	
7	Metachronous	Lung	M	M	0.695	Y
		Cervix	P	P	0.666	
10	Metachronous	Lung	M	M	0.715	Y
		Cervix	P	P	0.713	
12	Metachronous	Lung	P	P	0.259	Y
		Colon	P	P	0.647	
13	Synchronous	Lung	P	P	0.289	Y
		Cervix	P	P	0.548	
14	Metachronous	Lung	P	P	0.249	Y
		Colon	P	P	0.797	
5	Synchronous	Lung	Inconclusive	M	0.531	/
		Stomach		P	0.720	
6	Synchronous	Lung	Inconclusive	P	0.297	/
		Colon		M	0.652	
8	Synchronous	Lung	Inconclusive	M	0.575	/
		Colon		P	0.554	
9	Synchronous	Lung	Inconclusive	P	0.491	/
		Colon		M	0.455	
15	Synchronous	Lung	Inconclusive	P	0.428	/
		Stomach		P	0.556	
11	Synchronous	Lung	P	Not available	/	Y
		Stomach	P	P	0.751	
16	Synchronous	Lung	P	Not available	/	/
		Cervix	M	Not available	/	

### Survival outcomes of patients with lung-specific CUPs

**Fig. 4:**
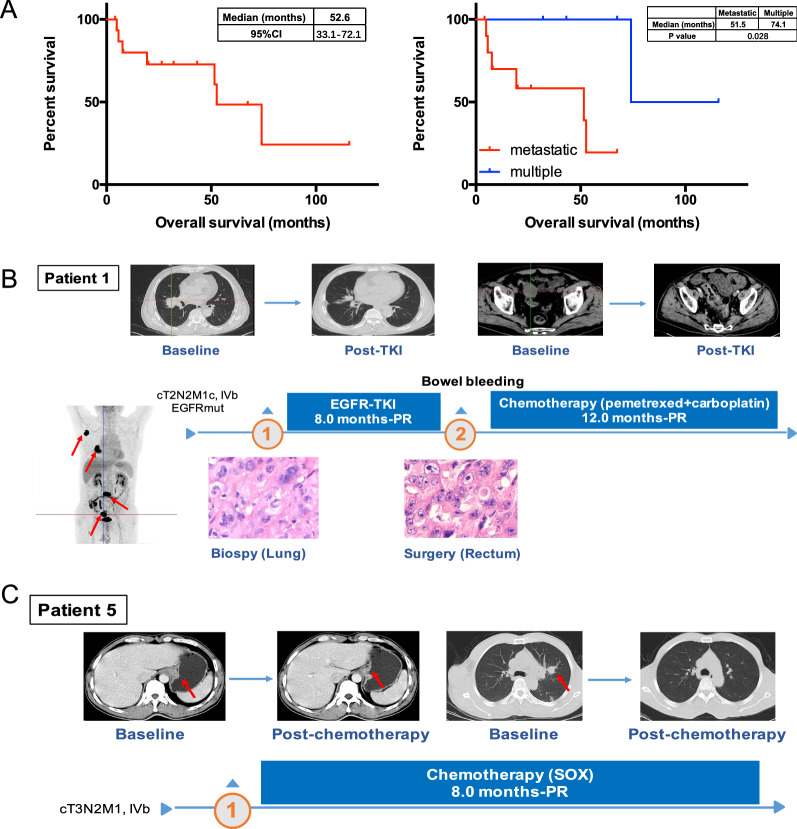
Survival outcomes and the courses of management of two cases. **A** Kaplan–Meier estimation of the overall survival (OS) curve for all 16 patients (left panel) and subgroups of patients predicted with metastatic cancer or multiple primary tumors (right panel). **B**, **C** Courses of management for two patients whose actual diagnoses coincided with our inferences. Patient 1 was diagnosed with lung cancer with colon metastasis and received first-line therapy with an EGFR tyrosine kinase inhibitor (TKI). Representative pulmonary and abdominal CT scans before and after TKI treatment are shown. Also shown are hematoxylin and eosin staining of lung biopsy and rectal surgical specimen, revealing histologic similarity between the two lesions. **C** Patient 5 was diagnosed and predicted with gastric cancer with lung metastasis. S-1 plus oxaliplatin (SOX) regimen was chosen accordingly, which has achieved favorable clinical response. CT: computed chromatography. PR: partial response

By-patient details of treatment regimens and survival outcomes are provided in Additional file 2: Table S1. After a median follow-up time of 67.4 months, the median OS was 52.6 months (95% confidence interval 33.1–72.1; Fig. 4A), which exceeded those reported in most studies of CUPs [11, 12]. As expected, patients diagnosed with metastatic disease had a significantly shorter OS (median 51.5 months) than those with multiple primary tumors (median 74.1 months; log-rank test p = 0.028; Fig. 4B), which attested to the accuracy of the CGP-time lag and DNA methylation analyses. TOO determination has significant therapeutic implications, which can be illustrated by the courses of management for patients 1 and 5. P1 was highlighted in Fig. 1B as a representative CUP case. The actual diagnosis coincided with our inference of lung cancer with colon metastasis (Table 2). The patient was treated with an EGFR inhibitor as first-line therapy based on axillary nodal disease and detection of activating *EGFR* mutations (Fig. 2B). After a PFS of 8 months and a best response of partial response (PR), he presented with bowel bleeding due to disease progression and underwent salvage surgery to remove the rectal lesion. Pemetrexed and carboplatin wad then administered as second-line therapy due to its well-established efficacy in lung cancer was then administered as second-line treatment [26], which achieved a PFS of 12 months as of the latest follow-up. Had colon been identified as the tumor origin, the patient could have received a chemotherapeutic regimen for instead of EGFR inhibitor as first-line treatment. P5 was another case in which the actual and putative diagnoses agreed. As shown in Fig. 4C, the patient was diagnosed with gastric cancer with pulmonary metastasis and treated with S-1 plus oxaliplatin, to which he responded favorably with a best response of PR and a PFS of 8 months. Together, the favorable clinical response, along with the markedly different survival outcomes between putative metastatic and multiple primary patients, suggested promising clinical value of the two molecular analyses.

## Discussion

In this study, we set out to establish a multidisciplinary approach for TOO identification for lung-specific CUPs. We included 16 CUP patients and applied two molecular analyses in parallel to compare their capability of unambiguous inference and inter-method concordance (Fig. 2). The two methods achieved 100% concordance when TOO can be clearly identified with DNA methylation analysis (Table 2), which was further supported by survival outcomes (Fig. 4). Based on these promising results, we propose an integrative molecular approach for patients with lung-specific CUP.

**Fig. 5:**
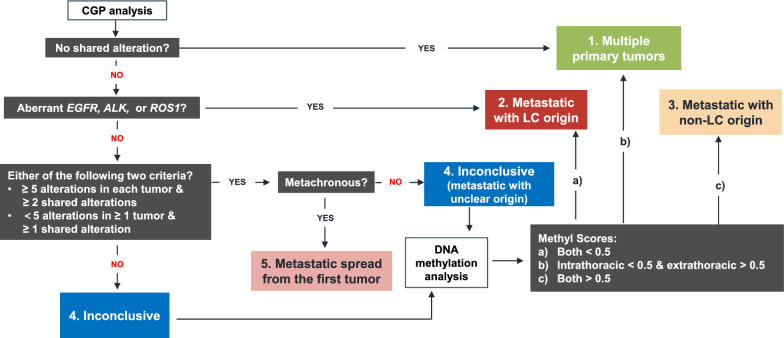
A schematic diagram of an integrative molecular approach for TOO identification for lung-specific CUP. Comprehensive genome profiling was first performed and subjected to a decision flow that yields five possible inferences, among which the inconclusive cases subsequently undergo DNA methylation analysis. Aberrant *EGFR, ALK* or *ROS1* refers to clinically actionable *EGFR* mutations or deletions or *ALK* or *ROS1* rearrangements detected in samples from both disease sites

As illustrated in Fig. 5, it is suggested that CGP be performed first, the resulting genomic profiles subjected a decision workflow and leading to one of four possible outcomes per the illustrated decision, and that DNA methylation be conducted only when CGP was inconclusive. This strategy was preferred over methylation profiling alone out of comprehensive consideration of relevant factors. A considerable proportion of lung-specific CUP patients carry tumors of pulmonary origin in the metastatic or the multiple primary setting, most of whom would undergo genomic profiling. Other cancer types such as colorectal and gastric cancer can also benefit from molecular testing that informs treatment biomarkers such as microsatellite stability, tumor mutation burden, and *HER2* amplification. Therefore, CGP would be suggested for a sizable of lung-specific CUP patients once methylation analysis determines the TOO. In addition, the CGP-time lag approach used in this study gave an impressive performance of unambiguous TOO inference for nearly 70% analyzed patients, all of whom had matching inferences by methylation analysis. Taking in these considerations, we placed CGP as a first step and methylation analysis as a need-based option to maximize cost effectiveness. A possible caveat of this strategy, however, is the additional turnaround time for methylation analysis for the patients who turn out in need of it. It is therefore suggested to establish a TOO-specific sequencing program that requires greater sample amount deposited and extracted for DNA in advance, and initiates methylation profiling once CGP data are found inconclusive, thereby eliminating the need for transportation and DNA extraction of a second tumor sample. Such a program would need greater sample amount deposited in advance. In our study, 30 ng of DNA was used for methylation, which was similar to the amount for CGP and would be feasible for most cases, since a median of 2710 ng DNA (range 370–6280) can be extracted from a 20-gauge core needle biopsy [27].

CGP has been used extensively in LC to distinguish multiple primary from intrapulmonary metastasis, which hinges on elucidation of tumor clonality [13, 14]. Compared with smaller panels, large gene panels could reduce the chance of assigning clonality to tumor pairs that shared alteration by chance. Indeed, using panels of up to 468 genes, a study showed that the odds of sharing hotspot mutations was practically nil between different primary lung tumors [13]. In this work, we applied a larger panel that targeted 520 genes to differentiate intra- and extrathoracic tumors. As expected, CGP alone was able to result in unambiguous inference of tumor clonality in all 16 (100%) patients and tumor origin for 7 (43.8%; i.e. Class I patients), including 4 with multiple primary and 3 with metastatic LC based on presence of highly specific LC drivers (actionable *EGFR* mutations: 2, *EML4-ALK* rearrangement: 1). Next, we designed the criterion of metachronous tumors based on the high propensity for metachronous tumors to result from metastatic spread [13], thereby incorporating clinical information typically available for CUP patients. This step led to TOO assignment for nearly half (4/9) of the remaining, undetermined cases. These results suggest the utility of integrating multidisciplinary evidence in tumor origin prediction.

The remarkable tissue specificity of epigenetic characteristics also been exploited for TOO prediction, which is featured by tissue origin classification based on DNA methylation patterns [28, 29]. In the EUICUP study, DNA methylation profiling predicted TOO in 87% (188/216) patients with CUP [15]. Patients treated with site-specific therapy showed significantly improved OS compared with those receiving empiric therapy. In our work, DNA methylation analysis achieved an even higher inference rate of 100%. Moreover, of the 9 patients assigned a tumor origin by both methods, CGP-time lag and DNA methylation analyses yielded matching results for all. To our knowledge, this study is the first to evaluate the concordance level between TOO determination via genome and DNA methylation profiling and report 100% concordance. Accuracy of TOO assignment was supported by the survival outcomes. Survival analysis showed significantly better OS in patients with putative multiple primary tumors than those with metastatic cancer. Additionally, we highlighted two cases in which the clinical responses to site-specific treatment corroborated our inferences.

Despite the promising results, this study has several limitations. First, despite the promising inter-analysis concordance and significantly improved OS, the small cohort size in this retrospective study warranted further validation of these findings. Also, as patients were treated with the physician’s choice of therapy, randomized studies are needed to characterize whether and how well the proposed integration could bring clinical benefits to CUP patients.

## Conclusion

In this study, we evaluated the performance and level of concordance between the two molecular analyses for TOO identification in CUP. Based on the promising results, we propose an integrative strategy that combines the two methods and clinical evidence. More clinical validation and randomized trials are warranted to further characterize the value of the proposed approach in managing lung-specific CUPs.

## Supplementary Information

**Additional file 1.** Comprehensive genomic profiling analysis and targeted methylation sequencing and data preprocessing.

**Additional file 2: Table S1. **Treatment regimens and survival outcomes for each of the 16 lung-specific CUP patients analyzed in this study.

## Data Availability

The datasets used and/or analyzed during the current study are available from the corresponding author on reasonable request.
