# Supplementary material for: A molecular approach integrating genomic and DNA methylation profiling for tissue of origin identification in lung-specific cancer of unknown primary
Source: J Transl Med. 2022 Apr 5;20:158. doi: 10.1186/s12967-022-03362-2 (PMC8981640; doi:10.1186/s12967-022-03362-2)
Supplement: Supplementary file 1 — Additional file 1. Comprehensive genomic profiling analysis and targeted methylation sequencing and data preprocessing. [file 12967_2022_3362_MOESM1_ESM.docx]

**Supplementary Methods**

**Comprehensive genomic profiling (CGP) analysis**

DNA isolation and targeted sequencing were performed in Burning Rock Biotech, a commercial clinical laboratory accredited by the College of American Pathologist (CAP) and certified by the Clinical Laboratory Improvement Amendments (CLIA), according to previously described procedures ^1, 2^. Briefly, tissue DNA was extracted from formalin-fixed, paraffin-embedded (FFPE) tumor tissues using QIAamp DNA FFPE tissue kit (Qiagen, Hilden, Germany). Fragments between 200–400 bp from the sheared tissue DNA were purified (Agencourt AMPure XP Kit, Beckman Coulter, CA, USA), hybridized with capture probes baits, selected with magnetic beads, and amplified. Target capture was performed using a targeted panel of 520 cancer-related genes (OncoScreen Plus, Burning Rock Biotech). The quality and the size of the fragments were assessed by high sensitivity DNA kit using Bioanalyzer 2100 (Agilent Technologies, CA, USA). Genomic DNA was then extracted from all samples, with matched leukocytes as germline DNA control. Indexed samples were sequenced on Illumina Nextseq 500 with paired-end reads and a target sequencing depth of 1,000×.

**Targeted methylation sequencing and data preprocessing**

Twenty-nine tumor tissue samples were sequenced using a capture-based bisulfite sequencing panel as described previously ^3^. The bisulfite sequencing (BS-seq) library was prepared using the brELSATM method (Burning Rock Biotech, Guangzhou, China). Briefly, purified DNA was treated with sodium bisulfite (EZ-96 DNA Methylation-Lightning™ MagPrep, Zymo Research, CA, USA). Single-strand DNA molecules were then ligated to a splinted adapter and amplified by a uracil-tolerating DNA polymerase to generate whole-genome BS-seq libraries. Custom-designed methylation profiling RNA baits were used for target enrichment which covers 80,672 CpG sites and spans 1.05 mega base of human genome. The target libraries were subsequently quantified by real-time PCR (Kapa Biosciences, MA, USA) and sequenced on NovaSeq 6000 (Illumina, CA, USA) with an average sequencing depth of 1,000X.

Removal of custom adaptor sequences and low-quality bases was performed with Trimmomatic. Afterwards, paired-end reads were aligned to C to T- and G to A-transformed hg19 genome with BWA-meth ^4^. We then removed duplicate reads with samblaster ^5^ and low mapping quality (MAPQ<20) or improper pairing reads with sambamba ^6^. Paired reads were merged by clipping overlapping reads to avoid double-counting of methylation calls. Methylation blocks were defined as the genomic region consisting of the neighboring CpG sites that were closely located and correlated in terms of methylation level.

**Supplementary Figures**


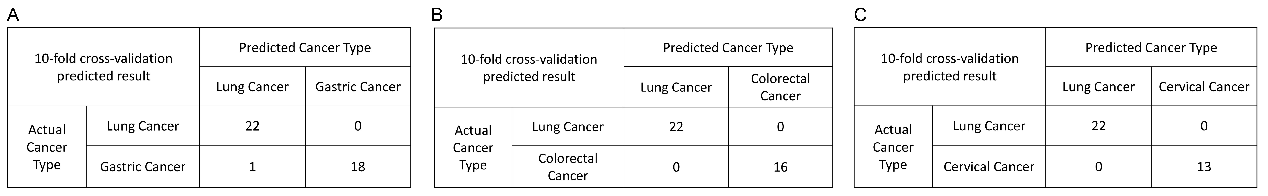


Figure S1. Result of cross-validation of tissue of origin predictions based on DNA methylation profiles.


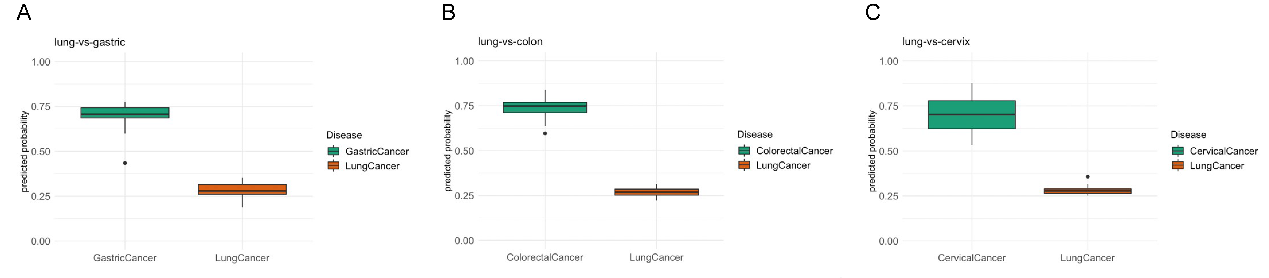


Figure S2. Distribution of probability scores produced by the three tissue of origin prediction models based on DNA methylation profiles.

**References**

1. Mao X, Zhang Z, Zheng X, et al. Capture-Based Targeted Ultradeep Sequencing in Paired Tissue and Plasma Samples Demonstrates Differential Subclonal ctDNA-Releasing Capability in Advanced Lung Cancer. *J Thorac Oncol* 2017;12:663-672.

2. Li YS, Jiang BY, Yang JJ, et al. Unique genetic profiles from cerebrospinal fluid cell-free DNA in leptomeningeal metastases of EGFR-mutant non-small-cell lung cancer: a new medium of liquid biopsy. *Ann Oncol* 2018;29:945-952.

3. Xia S, Ye J, Chen Y, et al. Parallel serial assessment of somatic mutation and methylation profile from circulating tumor DNA predicts treatment response and impending disease progression in osimertinib-treated lung adenocarcinoma patients. *Transl Lung Cancer Res* 2019;8:1016-1028.

4. Pedersen BS, Eyring K, De S, et al. Fast and accurate alignment of long bisulfite-seq reads. *arXiv:14011129v2 [q-bioGN] 2014* 2014.

5. Faust GG, Hall IM. SAMBLASTER: fast duplicate marking and structural variant read extraction. *Bioinformatics* 2014;30:2503-2505.

6. Tarasov A, Vilella AJ, Cuppen E, et al. Sambamba: fast processing of NGS alignment formats. *Bioinformatics* 2015;31:2032-2034.
